# Direct Reprogramming of Rat Neural Precursor Cells and Fibroblasts into Pluripotent Stem Cells

**DOI:** 10.1371/journal.pone.0009838

**Published:** 2010-03-24

**Authors:** Mi-Yoon Chang, Dohoon Kim, Chun-Hyung Kim, Hoon-Chul Kang, Eungi Yang, Jung-Il Moon, Sanghyeok Ko, Junpil Park, Kyung-Soon Park, Kyung-Ah Lee, Dong-Youn Hwang, Young Chung, Robert Lanza, Kwang-Soo Kim

**Affiliations:** 1 Molecular Neurobiology Laboratory, McLean Hospital, Harvard Medical School, Belmont, Massachusetts, United States of America; 2 Harvard Stem Cell Institute, Cambridge, Massachusetts, United States of America; 3 CHA Stem Cell Institute, Pochon CHA University College of Medicine, Seoul, Korea; 4 Stem Cell International, Inc., Worcester, Massachusetts, United States of America; University of Washington, United States of America

## Abstract

**Background:**

Given the usefulness of rats as an experimental system, an efficient method for generating rat induced pluripotent stem (iPS) cells would provide researchers with a powerful tool for studying human physiology and disease. Here, we report direct reprogramming of rat neural precursor (NP) cells and rat embryonic fibroblasts (REF) into iPS cells by retroviral transduction using either three (Oct3/4, Sox2, and Klf4), four (Oct3/4, Sox2, Klf4, and c-Myc), or five (Oct3/4, Sox2, Klf4, c-Myc, and Nanog) genes.

**Methodology and Principal Findings:**

iPS cells were generated from both NP and REF using only three (Oct3/4, Sox2, and Klf4) genes without c-Myc. Two factors were found to be critical for efficient derivation and maintenance of rat iPS cells: the use of rat instead of mouse feeders, and the use of small molecules specifically inhibiting mitogen-activated protein kinase and glycogen synthase kinase 3 pathways. In contrast, introduction of embryonic stem cell (ESC) extracts induced partial reprogramming, but failed to generate iPS cells. However, when combined with retroviral transduction, this method generated iPS cells with significantly higher efficiency. Morphology, gene expression, and epigenetic status confirmed that these rat iPS cells exhibited ESC-like properties, including the ability to differentiate into all three germ layers both in vitro and in teratomas. In particular, we found that these rat iPS cells could differentiate to midbrain-like dopamine neurons with a high efficiency.

**Conclusions/Significance:**

Given the usefulness of rats as an experimental system, our optimized method would be useful for generating rat iPS cells from diverse tissues and provide researchers with a powerful tool for studying human physiology and disease.

## Introduction

The cloning of Dolly the Sheep over a decade ago demonstrated that adult somatic cells could reprogrammed back to a state of pluripotency [1]. In 2006, Yamanaka and his colleagues showed that retroviral transduction of four transcription factors (Oct4, Sox2, Klf4 and c-Myc) could also induce pluripotency in mammalian (mouse) cells [2]. Subsequent studies demonstrated that human induced pluripotent stem (hiPS) cells could be generated using the same or slightly different sets of reprogramming factors, offering the possibility to generate disease- or patient-specific stem cells [3], [4], [5], [6], [7], [8], [9], [10].

The rat animal model is one of most valuable models for the study of numerous human diseases as well as for therapeutics development. For instance, 6-OHDA lesioned rats is one of most popular animal model for Parkinson's disease (PD) [11], [12], [13]. Notably, however, its biological and biomedical study is limited because the generation of transgenic rats by targeted gene manipulation is not yet established. Recently, three groups reported the establishments of chimera- and/or germline-competent ESCs from rat blastocysts [14], [15], [16], strongly suggesting that it will be possible to generate transgenic rats by targeted gene manipulation in the near future. In addition, two groups recently reported generation of iPS cells from rat liver progenitor cells [17] or primary ear fibroblasts and bone marrow cells [18].

In this study, we sought to establish an efficient procedure to generate iPS cells from two different rat tissues, neural precursors (NPs) and rat embryonic fibroblast (REF), by introducing total extracts from ESCs and/or retroviral transduction of defined transcription factors. We found that introduction of ESC-extracts into rat NP cells failed to generate iPS cells inducing only partial reprogramming. However, rat iPS cells were successfully generated from both NPs and REF by retroviral transduction of reprogramming factors with or without c-Myc, and the efficiency was significantly improved when these two methods were combined. Notably, we established an optimal procedure to generate and maintain rat iPS cells by culturing the cells on REF instead of mouse embryonic fibroblast (MEF) as the feeder in the presence of mitogen-activated protein kinase kinase (MEK) and glycogen synthase kinase 3 (GSK3β) inhibitors (PD0325901 and CHIR99021, respectively). Rat iPS cells derived from our optimized procedure exhibited ESC-like properties by morphological, gene expression, epigenetic status, proliferation, and differentiation criteria. In particular, we show that these rat iPS cells can efficiently differentiate to multiple neuronal lineages including midbrain-like dopaminergic neurons which will serve as invaluable platform for bioassay and cell transplantation studies of PD.

## Materials and Methods

### Cell Culture

We employed neural precursor (NP) cell culture from micro-dissected cortices from rat embryonic day 14 (day of conception = day 0). Time-pregnant Sprague-Dawley (SD) rats were purchased from Charles River Laboratories. INC. (Wilmington, MA). All animal procedures were performed in accordance with National Institute of Health guidelines and were approved by the Animal Care and Use Committee (IACUC) at McLean Hospital, Harvard Medical School.

Embryonic cortices were dissected from rat embryos and mechanically dissociated in Ca^2+^/Mg^2+^-free Hank's balanced salt solution (CMF-HBSS). Cells were plated at 8000 cells/cm^2^ on 10 cm tissue culture dishes pre-coated with poly-L-ornithine (PLO; 15 µg/ml) at 37°C two hours followed by fibronectin (FN; 1 µg/ml) overnight. NPs were allowed to proliferate in the presence of 20 ng/ml basic fibroblast growth factor (bFGF; R&D Systems, Minneapolis, MN) in serum-free medium (N2) for 4–6 days [19], [20]. For rat embryonic fibroblast (REF) isolation, uteri isolated from 14-day-pregnant SD rats were washed with phosphate-buffered saline (PBS). The head and visceral tissues were removed from isolated embryos. The remaining bodies were washed in fresh PBS, transferred into a 0.1 mM trypsin/1 mM EDTA solution, and incubated for 20 min. After incubation, REF culture medium (DMEM containing 15% defined FBS) was added and cells were dissociated by pipetting. We used REFs at passage two for reprogramming experiments and for feeders. For mouse embryonic fibroblast (MEF) cells, 13-day-pregnant CD1 mice were used under the same isolation and culture methods.

Induced pluripotent stem (iPS) cells were generated and maintained in ES medium, Dulbecco's modified Minimal Essential Medium (DMEM, Invitrogen, Carlsbad, CA), supplemented with 2mM L-glutamine (Invitrogen, Carlsbad, CA). 1mM β-mercaptoethanol, 1x non-essential amino acids (NEAA; Invitrogen, Carlsbad, CA), 15% fetal bovine serum (FBS, Sigma-Aldrich, St. Louis, MO), 100 U/ml penicillin, 100 µg/ml streptomycin (Invitrogen) and 2000 U/ml leukemia inhibitory factor (LIF; Chemicon, Termecula, CA) supplemented with signal inhibitors, CHIR99021 (3 µM; Axon Medchem, Groningen, Netherland) and PD0325901 (0.5 µM; Axon Medchem). IPS cells were maintained on feeder layers of mitomycin C (10 µg/ml media, Sigma-Aldrich)-treated REF cells. For picking and passaging, rat iPS cells were washed once with ES medium and then mechanically picked (until passage 10) or incubated with 1 mg/ml collagenase type IV (Stem cell Technology, INC., Vancouver, Canada) for 10 min. An appropriate volume of the medium was added, and the contents were transferred to a new dish on REF feeder cells. The split ratio was 1∶1 (until passage 5) and after routinely 1∶3. For feeder-free culture of iPS cells, the plate was coated with gelatin (Stem cell Technology, INC.).

### Making cell extracts and streptolysin O (SLO)-mediated permeabilization and cell extract treatment

Mouse ESCs (J1) were propagated in vitro using feeder-free conditions without signal inhibitor supplementation. ESC-extracts were prepared when cultures reached 70–80% confluence. To prepare ESC-extracts, cells were washed with PBS once followed by one wash with cell lysis buffer (100 mM HEPES, pH 8.2, 50 mM NaCl, 5 mM MgCl2, 1 mM dithiothreitol, and protease inhibitors), followed by sedimentation at 400 g, suspension in 1 volume of cold cell lysis buffer, and incubated for 30–45 min on ice. Cells were sonicated. The supernatant was aliquoted, frozen and stored at −80°C. Lysate from 10 million J1-ESCs was used to generate 100 µl of extract. Control NP or pluripotent factors infected NP cells were washed in cold PBS and in cold CMF-HBSS. Cells were suspended in aliquots of 100,000 cells/100 µl of HBSS, and centrifuged at 2,500 g for 5 min at 4°C. Sedimented cells were suspended in HBSS, and streptolysin-O (SLO; Sigma) was added to a final concentration of 400 ng/ml. Permeabilization was assessed by monitoring uptake of FITC-labeled F(ab')2-antibodies from a separate sample 24 h after resealing and replating the cells. After permeabilization, cells were suspended at 1000 cells/µl in 100 µl of ESCs extract containing an ATP-regenerating system (1 mM ATP, 10 mM creatine phosphate, and 25 µg/ml creatine kinase), 100 µM GTP (Sigma-Aldrich), and 1 mM each nucleotide triphosphate (NTP). The tube containing cells was incubated horizontally for 1 h at 37°C in a CO_2_-incubator with occasional agitation. After dissociation, cells were plated into gelatin-coated plates for clone formation with N2/ES media (1∶1 volume mixture) containing bFGF (1 ng/ml). To reseal plasma membranes, we add 2 mM CaCl_2_ to the culture media.

### Retroviral infection

The cDNA encoding hOct4, hSox2, hKlf4, hNanog and hc-Myc (Open Biosystems) were subcloned into the pCL retroviral expression vector [21]. The nucleotide sequences for the cloned genes were confirmed by sequence analysis. Each recombinant plasmid was introduced into the 293gpg retrovirus packaging cell line [21] by transient transfection, followed by harvesting.

For viral transduction, NP or REFs cultured *in vitro* were incubated with the viral supernatant containing polybrene (hexadimethrine bromide; 1 µg/ml; Sigma) overnight. After infection, cells were incubated six more days in ES culture media. At day six of infection, cells were replated on REF-feeder plate with or without ESC-extracts treatment.

### RT-PCR analysis for marker genes

Total RNA was purified with Trizol reagent (Invitrogen), five micrograms of total RNA were used for reverse transcription reaction with SuperScript II (Invitrogen) and oligo-dT primer, according to the manufacturer's instructions. PCR reaction conditions were optimized to determine the linear amplification range. Amplification products were identified by size. Primer sequences were: glyceraldehyde-3-phosphate dehydrogenase (GAPDH)

(5′-TGACATCAAGAAGGTGGTGAAGC-3′, 5′-CCCTGTTGCTGTAGCCGTATTG-3′); endogenous rat specific Oct4 (5′-CCGAGGGCCAGGCAGGAGCACGAG-3′, 5′-CCCTGGGAAAGGTTTCCCGCG-3′); endogenous rat specific Nanog (5′-AGGGTCTGCTACTGAGATGCTCTG-3′, 5′-AGGTCTGACTGCCCCATACTGG-3′); transgenic Oct4 (5′-GAAGGATGTGGTCCGAGTGT-3′, 5′-CATCTGTTCTTGGCCCTGAG-3′); transgenic Klf4 (5′-CCCACACAGGTGAGAAACCT-3′, 5′-CATCTGTTCTTGGCCCTGAG-3′); transgenic Sox2 (5′-TACCTCTTCCTCCCACTCCA-3′, 5′-CATCTGTTCTTGGCCCTGAG-3′); embryonic cell-activated transcpript 1 (Ecat1) (5′-AGGTCAACGAGGCTGCCA-3′, 5′-GGGTCTCCCACTCAAAAACC-3′); embryonic cell-specific gene 1 (ESG1) (5′-TCCAGAAGTATTCCAGGTCCA-3′, 5′-CTCCAGGGTCTTCATGGATT-3′); reduced expression 1 (REX1) (5′-AGGATGGCCGGAAGGAGAA-3′, 5′-TGCCCGTCCACATTGTCTT-3′); fibroblast growth factor 4 (FGF4) (5′-CAGCGGGGCAGGGGACTA-3′, 5′-CTTGGTCCGCCCGTTCTTAC-3′); forkhead box A2 (FOXA2) (5′-GCAAGCAAGGAAGCCTATCTT-3′, 5′-GGTGCAGCACTGATCTACAA-3′); Brachyury (5′-AGTACGAACCTCGGATTCAC-3′, 5′-CTGAGACTTGTAGACAACTGG-3′); and bIII-tubulin (Tuj1) (5′-TGGACAGTGTTCGGTCTGG-3′, 5′-CCTCCGTATAGTGCCCTTTGG-3′).

### Alkaline phosphatase staining and immunocytochemistry

Alkaline phosphatase (AP) staining was performed using the Alkaline phosphatase staining kit II (Vector Vector Laboratories, Burlingame, CA).

For immunocytochemistry, cells were fixed with 4% paraformaldehyde for 20 min at room temperature. After washing with PBS, cells were treated with PBS containing 10% normal goat serum and 0.1% Triton X-100 for 35 min at room temperature. Primary antibodies included SSEA1 (monoclonal, 1∶100, Developmental Studies Hybridoma Bank, Iowa, IA), Nanog (polyclonal, 1∶300, Abcam, Cambridge, MA), Rex1 (polyclonal, 1∶200, Abcam), smooth muscle actin (SMA; monoclonal, 1∶400, Dako, Glostrop, Denmark), anti-βIII tubulin (Tuj1; monoclonal, 1∶500, Covance, Richmond, CA), GFAP (polyclonal, 1∶500, DAKO), Sox17 (monoclonal, 1∶200, SantaCruz Biotech.), tyrosine hydroxylase (TH; rabbit or sheep polyclonal,1∶1,000, Pel-Freez, Rogers, AR), serotonin (5-HT; polyclonal,1∶2000, Sigma), GABA (polyclonal, 1∶700, Sigma), choline acetyl transferase (ChAT; polyclonal, 1∶700, Sigma), nestin (monoclonal, 1∶1,000, BD Sciences, Franklin Lakes, NJ), Ki67 (monoclonal, 1∶500, Novocastra laboratories Ltd., United Kingdom), Ptx3 (polyclonal, 1∶200, Zymed), Engrailed-1 (En-1; monoclonal, 1∶100, Developmental Studies Hybridoma Bank), collagen type I (monoclonal, 1∶100, Developmental Studies Hybridoma Bank), Fibronectin (monoclonal, 1∶5, Developmental Studies Hybridoma Bank). For detection of primary antibodies, fluorescence-labeled (Alexa fluor 488 or 568; Molecular Probes, Eugene, OR) secondary antibodies were used according to the specifications of the manufacturer. Cells were mounted in Vectashield containing 4′,6-diamidino-2-phenylindole (DAPI; Vector Lab.) and analyzed under a fluorescent microscope.

### In vitro differentiation of iPS cells

Cells were harvested by trypsinization and transferred to bacterial culture dishes in ES medium without LIF. Total RNA derived from EB on day 4 was used for RT-PCR analysis. After 5 days, aggregated cells were plated onto tissue culture dishes and incubated for another 8∼10 days with serum-free ITSFn medium [22]. These differentiated cells were stained with antibodies specific for the three germ layers. For neural induction, the 5-staged method [22] was used with simple modifications. Briefly, iPS cells were dissociated and EBs were allowed to form for five days after plating of iPS cells in bacterial dishes in ES medium without LIF (EB media; stage 2). EBs were attached to tissue culture dishes and neural precursor were then selected for by incubation in serum-free ITSFn medium (DMEM/F12 media containing apotransferrin (50 µg/ml), insulin (5 µg/ml), sodium selenite (30 nM), fibronectin (250 ng/ml), 100 U/ml penicillin, 100 µg/ml streptomycin ) for 4–6 days (stage 3). Cells were subsequently dissociated by trypsin (0.05%) and neuronal precursors expanded and patterned for four days after plating onto PLO/FN-coated plates at a density of 50,000 cells/cm2 in DMEM/F-12 media with apotransferrin (100 µg/ml), insulin (5 µg/ml), sodium selenite (30 nM), progesterone (20 nM), putrescine (100 nM), 100 U/ml penicillin, 100 µg/ml streptomycin, 1 µg/ml laminin (N3 media) with bFGF (20 ng/ml), at day 3, Shh (500 ng/ml) and FGF8 (100 ng/ml ; all from R & D Systems) were added (stage 4). The cells were subsequently differentiated in N3 media with ascorbic acid (AA; 200 µM) for 7 days (stage 5).

### Teratoma formation

Rat NP-iPS cells (#2 and #4) and RES-iPS cells (#3 and #4) were suspended in DMEM containing 10% FBS. Nude mice were anesthetized and the cell suspension was injected subcutaneously into the kidney capsule. Four to six weeks after the injection, tumors were surgically dissected from the mice. Samples were weighed, fixed in PBS containing 4% formaldehyde, and embedded in paraffin. Sections were stained with hematoxylin and eosin.

### Bisulfite genomic sequencing

Genomic DNA from cells was performed with the DNeasy Tissue Kit (Qiagen, Valencia, CA). Bisulfite treatment was done using the EpiTect Kit (Qiagen) following the manufacturer's instruction. Bisulfite treated DNA was amplified using primers designed for methylation PCRs (http://www.urogene.org//methprimer/index.html): the forward primer 5′-AGT TTT GAG GTG TTT AGG GAT TTA T-3′ and reverse primer 5′-CCC CAC CAA ATA AAA ATA AAA AAA-3′ for Oct4, and the forward primer 5′-GGG TTT GGT AGG AGG GAT TAA T-3′ and reverse primer 5′-TCA ACC TAT CTA AAA ACC AAC AAC TC-3′ for Nanog. For more products, semi-nested PCR was performed using the forward primer 5′-GAA AAT GAA GGT TTA TTT GGT TGT-3′ for Oct4 and 5′-TTT GGT AGG AGG GAT TAA TTG TG-3′ for Nanog, respectively and in reverse as above. The resulting amplified PCR products were gel-purified, subcloned into the T vector (Promega, Madison, WI) and sequenced.

### Karyotyping analysis

Standard G-band chromosome analysis was performed by Cell Line Genetics (Madison, WI).

## Results and Discussion

### Introduction of ESC-extracts into rat NPs resulted in partial reprogramming but failed to generate iPS cells

Based on previous studies showing that fusion of ESCs with somatic cells induce nuclear reprogramming [23], [24], [25], we hypothesized that introduction of ESC total proteins into rat somatic cells may reprogram them to a pluripotency. To address this, we attempted to generate rat iPS cells by introducing total ESC-extracts into permeabilized rat NP cells. Toward this goal, we dissected NPs from E14 rat cortices and expanded them in the presence of basic-fibroblast growth factor (bFGF). The great majority of these expanded NP cells (>98%) were positive for nestin and the active mitotic marker Ki67 (Figure S1). 5×10^4^ NP cells were treated with mouse ESC-extracts following streptolysin O-mediated permeabilization. Fifteen days following ESC extract treatment, approximately 10 colonies appeared, while no colonies were observed in the absence of treatment or when treated with 293T cell extracts. These colonies were flat, adherent and non-granulated (Figure S2a). When they were picked up and replated onto mitomycin C-treated mouse embryonic fibroblast (MEF) feeders, they did not exhibit large nucleoli and appeared different from typical dense ESC-like morphology. Approximately 20% of these clones expressed Nanog, but none of them expressed other pluripotent markers such as Oct4 or were positive for alkaline phosphatase (AP) (Figure S2b; data not shown). Following maintenance under neural culture condition and in vitro differentiation, these clones could generate neurons and astrocytes (Figure S2c). In contrast, NP cells that were maintained without ESC extract treatment mostly differentiated into astrocytes (Figure S2c, d). Together, our results show that introduction of ESC-extracts into permeabilized NP cells induced partial reprogramming but failed to generate iPS cells.

### Generation of ESC-like iPS cells from rat NPs and fibroblasts

**Figure 1:**
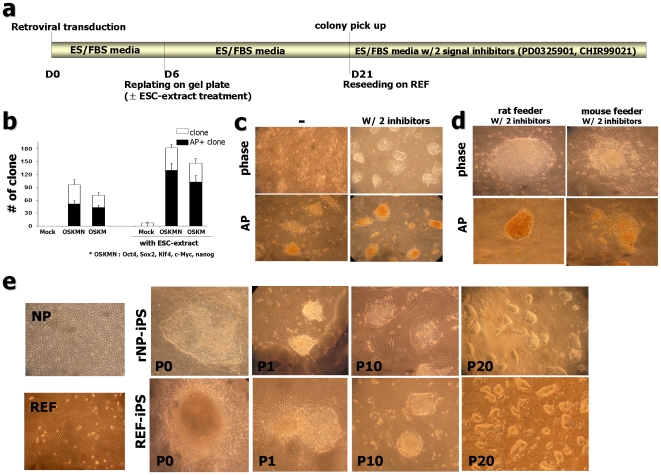
Generation of iPS cells from rat neural precursor cells (rNP) and embryonic fibroblast cells (REF). (a) Schematic time schedule of rat-iPS generation. (b) Clone formation after pluripotent factors induction with or without ESC-extract treatment. Shown are alkaline phosphatase (AP)-positive clone numbers (black columns) from the total numbers of colony (white columns) at 20 days after transduction. (Each column from n = 14 of 6 independent experiments, error bars indicate S.E.; *OSKMN: **O**ct4, **S**ox2, **K**lf4, c-**M**yc, **N**anog) (c) Colonies with ESC morphology could be maintained in the presence of MEK inhibitor, PD0325901 (0.5 µM) and GSK3β inhibitor, CHIR99021 (3 µM) (right). Colonies under conventional ES culture condition lost AP-positive character and kept differentiating (left). (d) Rat embryonic fibroblast (REF)-derived feeders are necessary for maintaining ESC-like character. After several passages, ESC-like colonies lost AP activity on mouse embryonic fibroblast (MEF)-derived feeders combined with 2 inhibitors, PD0325901 and CHIR99021. (e) Phase-contrast micrograph of rat embryonic NP and fibroblast (far left). Isolation of rNP-iPS and REF-iPS cells based on morphology, pictures on the right show rNP-iPS #2 clone and REF-iPS #3 formation, respectively. P0 image shows the representative clone formed at 20 days after infection (P0: passage 0) and image P1 was taken at 7 days after picking (passage 1). Examples of homogeneous colonies from images on the left at passage number 10 (P10) and on the right at passage number 20 (P20).

Next, we tested if retroviral transduction of the five (Oct4, Sox2, Klf4, c-Myc, and Nanog: OSKMN) and/or four factors (OSKM) could reprogram rat NPs and fibroblasts to generate iPS cells. Monitoring infection efficiency with the GFP-expressing pCL-retroviral vector, revealed that the majority of rat NPs (>95%) could be transduced (data not shown). The time schedule for rat iPS cell induction is summarized in Figure 1a. First, we examined the formation of colonies after retroviral transduction to express the five or four factors. Six days post retroviral transduction NPs were replated on mitomycin C-treated feeder cells. For control, the same titer of pCL empty virus or GFP-expressing virus was used for transduction. Colonies started to appear from five and four factor-transduced NPs at day 13 to 16 post-infection, but not from empty vector-or GFP vector-transduced cells. These colonies were highly proliferative and adopted a morphology distinct from those formed by ESC extract treatment (Fig. 1c). Some of these clones exhibited granulation and AP activity. We first tested if rat NP-derived ESC-like colony formation is influenced by feeder variants, i.e., MEF vs. rat embryonic fibroblast (REF) feeders. Interestingly, we found that AP-positive colonies were generated with significantly higher efficiency on REF than on MEF. For instance, 51 AP-positive colonies (out of total 93 colonies) were formed from 5×10^4^ NPs by treatment with 5 factors on REF, while 36 AP+ colonies (out of total 53 colonies) were formed on MEF feeder (Figure S3). In the case of 4 factors (OSKM) transfection, 43 AP-positive colonies (out of 67 total colonies) and 20 AP-positive (out of 34 total colonies) were formed on REF and MEF, respectively. Approximately 20% of these AP-positive colonies exhibited ESC-like morphology and approximately 40% of AP-positive clones were SSEA1 positive (Figure S4B; data not shown).

We next tested whether retroviral transduction of 3 factors (Oct4, Sox2, and Klf4: OSK) without c-Myc can also generate ESC-like colonies. Although the efficiency was lower than 5 and 4 factors transduction, AP-positive ESC-like colonies could be generated by 3 factors without c-Myc (Figure S3). Again, REF was significantly better than MEF for generation of reprogrammed colonies by 3 factors. Furthermore, we could generate ESC-like colonies by 5, 4, and 3 factor transduction from REF cells as the starting cells (Figure S4). Interestingly, similar to generation of ESC-like colonies from NP cells, REF was significantly better than MEF as feeder for generation of ESC-like colonies from REF (data not shown). Thus, in this case, rat embryonic fibroblasts were used as both starting cells and feeders for reprogramming.

Notably, these NP or REF-derived ESC-like colonies lost ESC-like morphology and AP activity when maintained under conventional murine ESC culture condition (Fig. 1c, left). Based on recent studies showing that inhibition of differentiation inducing signals is essential for derivation and establishment of rat ESCs [14], [15], we speculated that the same treatment may be necessary for rat iPS cell maintenance. Indeed, we found that treatment with MEK inhibitor (PD0325901; 0.5 µM) and GSK3β inhibitor (CHIR99021; 3 µM) significantly improved the maintenance of both NP- and REF-derived ESC-like cells for their morphology and AP activity (Fig. 1c, right). Notably, even in the presence of these two inhibitors, these NP or REF-derived ESC-like cells subsequently differentiated and lost their ESC-like morphology on MEF feeders (Fig. 1d), again emphasizing the importance of REF as feeder cells.

Using our established procedure, we further tested if combination of retroviral transduction and ESC extract treatment can improve the efficiency of reprogramming. Toward this end, NPs were permeabilized and treated with ESC-extracts six days post retroviral transduction (Fig. 1a). Interestingly, the efficiency to generate AP-positive colonies was significantly higher (Fig. 1b). When retroviral transduction was combined with ESC-extracts treatment, the efficiency AP-positive colonies was >0.2%, in the case of five factor transduction plus ESC extract treatment (Fig. 1b). Notably, this combined treatment resulted in accordingly enhanced generation of ES-like morphology clones (>2.5-fold); while 9 ES-like colonies out of 48 AP+ clones were generated by retroviral four factor transduction, 24 ES-like colonies out of 102 AP+ clones were formed by combined treatment of retroviral 4 factor transduction and ESC-extract treatment (data not shown).

### Putative rat iPS cells exhibit ESC-like properties in proliferation, gene expression of ESC markers, and epigenetic status

**Figure 2:**
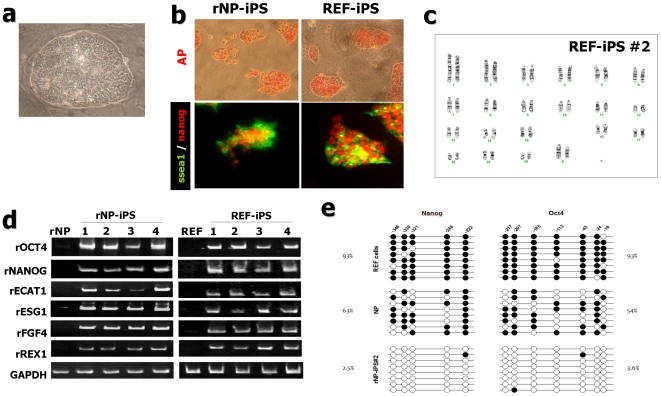
Rat neural precursor cell and fibroblast derived-iPS cells (rNP-iPS and REF-iPS) share ESC characters. (a) Representative high magnified image of rat iPS cells grown on feeder. (b) Both rNP-iPS and REF-iPS cells exhibited strong alkaline phosphatase (AP) activity and were homogeneously labeled with antibodies against SSEA1 (green) and Nanog (red). (c) No karyotypic abnormalities were observed in REF-iPS #2. (d) iPS clones derived from rat neural precursor (rNP-iPS #1∼#4) and fibroblast (REF-iPS #1∼#4) express ESC markers. RT-PCR analysis of ES marker genes, Oct4, Nanog, ECAT1, ESG1, FGF4 and REX1. Rat neural precursor cell (rNP) and rat embryonic fibroblast cells (REF) were used as negative control. GAPDH was used as a loading control. (e) DNA methylation status upstream of Nanog and Oct4 in rat PC12, rat NP, and rNP-iPS clone (#2) using sodium bisulfite sequencing. The top panel indicates the CpG dinucleotide position of the Nanog and OCT4 promoter regions and the numbers show positions of CpGs relative to the translation start site. Each PCR product was subcloned and subjected to nucleotide sequencing analysis. Eight representative sequenced clones are depicted by open (unmethylated) and filled (methylated) circles for each CpG site.

For further analyses, we selected 11 iPS-like clones derived from NP (5 by OSKMN, 3 by OSKM, and 3 by OSK) and 9 clones derived from REF (3 by OSKMN, 2 by OSKM, and 4 by OSK)(Fig. 1e). These clones formed tightly packed colonies and exhibited morphology almost identical to that of murine ESCs, characterized by large nucleoli, scant cytoplasm and round shape (Fig. 2a). Among these clones, we cultured and propagated 4 NP-derived clones (rNP-iPS#1-#3 by OSKMN and rNP-iPS#4 by OSK) and 4 REF-derived clones (REF-iPS#1 by OSKMN, REF-iPS#2 by OSKM, REF-iPS#3, #4 by OSK) in ESC media supplemented with 2 inhibitors on REF feeders for more than 25 passages. During this process, ESC-like morphology and growth characteristics were consistently maintained in all 8 clones. These ESC-like morphological and proliferative properties have been maintained for at least 5 months with normal karyotypes (Fig. 2c, Figure S5). These results strongly suggest that stable rat iPS cell lines can be generated from rat NPs and REFs by retroviral transduction of 5 (OSKMN) and 4 (OSKM) factors, as well as 3 (OSK) factors without c-Myc. We next examined these iPS cell lines' ESC marker gene expression. Immunocytochemical analyses showed that all eight clones expressed endogenous ESC-specific markers such as AP, Nanog, the surface antigen SSEA-1 (Fig. 2b), and reduced expression 1 (REX1) (Figure S6a and data not shown). Furthermore, RT-PCR analysis revealed that these iPS clones expressed all rat endogenous ESC markers tested, including Oct4, Nanog, embryonic cell-activated transcript 1 (Ecat1), embryonic cell-specific gene 1 (ESG1), REX1, and fibroblast growth factor 4 (FGF4) (Fig. 2d), further supporting that these clones show marker gene expression pattern that is indistinguishable from ESCs. We next tested whether the exogenous retroviral transgenes are efficiently suppressed in these iPS cells. As shown in Figure S6b, six rat iPS clones tested by semi-quantitative PCR analyses using specific primers demonstrated that exogenous transgenes are undetectable or only marginally expressed while endogenous genes are robustly induced. These observations strongly suggest that retroviral-coded genes are efficiently suppressed in these rat iPS cells.

The above gene expression of ESC markers strongly suggests that epigenetic reprogramming has occurred in these rNP-iPS and REF-iPS cells. To address this possibility, we next investigated the epigenetic status of the Oct4 and Nanog gene promoters in rat iPS cells (Fig. 2e). In order to investigate the basic stable methylation pattern in rat somatic cells, we analyzed the methylation status of both loci by bisulfite sequencing. As shown in Fig. 2e, the promoter regions of Oct4 and Nanog genes were densely methylated in REF cells (93%), suggesting that their chromosomes represent the differentiated stage. Then we further performed bisulfite sequencing with genomic DNAs extracted from NPs and rNP-iPS cells. Interestingly, we found that promoter regions of rat Oct4 and Nanog were much less methylated in NP isolated from E14 rat cortex (54% and 63%, respectively). In rNP-iPS#2 cells, both promoter regions of the rat Oct 4 and Nanog genes were almost completely demethylated (3.6% and 2.5%, respectively).

### Rat iPS cells differentiate into all three germ layers both in vitro and in vivo

**Figure 3:**
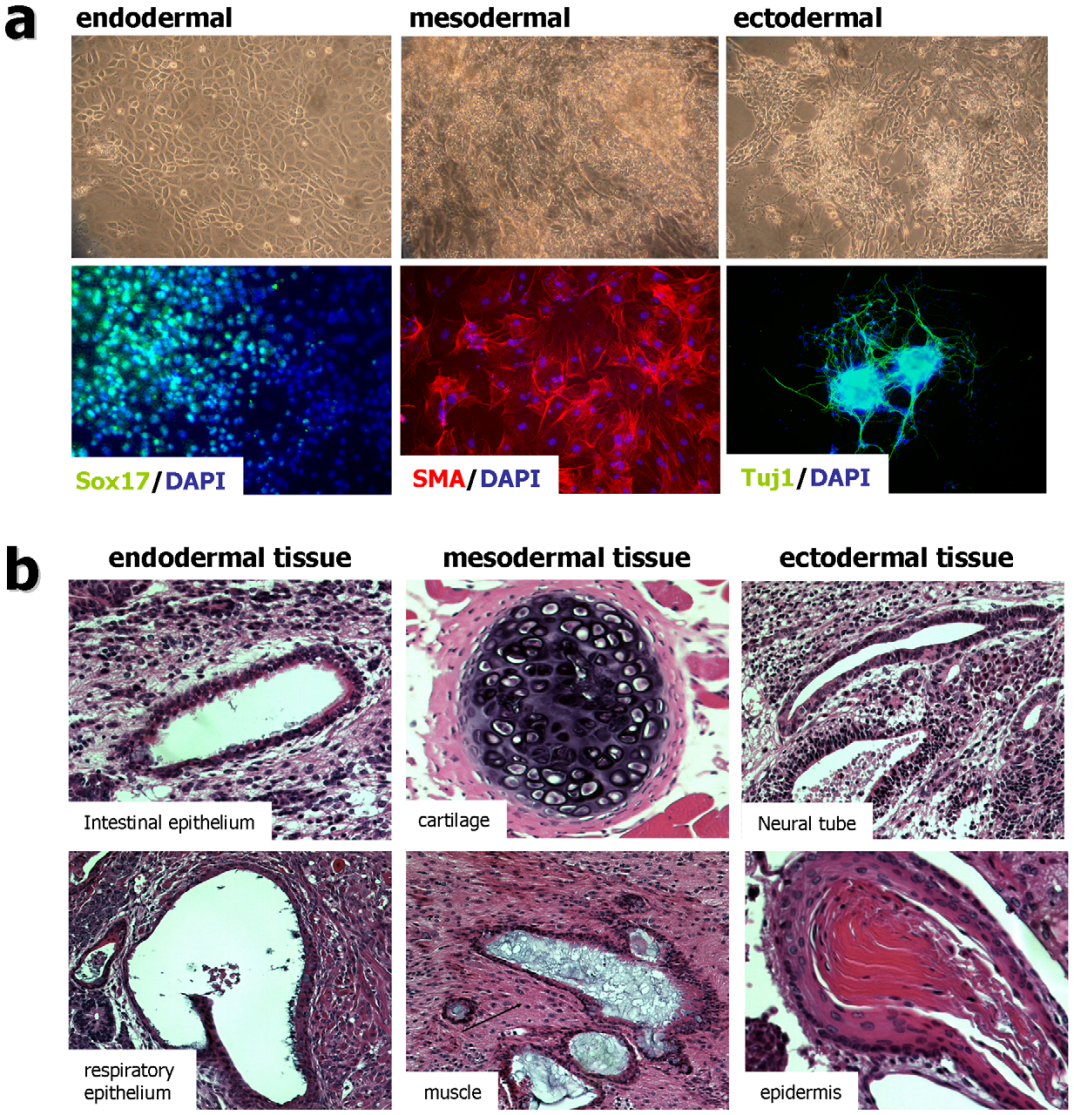
In-vitro and in-vivo differentiation of rat fibroblast derived iPS cells (REF-iPS #2 and #3). (a) Embryoid body (EB) mediated in vitro differentiation of rat iPS clones. Upper panel show phase contrast images of differentiated cells at 10 days after EB attachment. Definitive endoderm-like (left), contracting muscle-like (middle) and neuronal-like (right) cells are shown. Immunocytochemical analysis for the three germ layer differentiation was performed 10 days after EB attachment (Lower images). Sox17 (green, endodemal; left), smooth muscle actin (SMA, red, mesodermal; middle) and Tuj1 (green, ectodermal; right). Nuclei were stained with DAPI (blue). (b) Hematoxylin and eosin staining of teratoma derived from rFC-iPS cells (#2 and #3). Cells were transplanted into the kidney capsule of three SCID mice. A tumor developed from one injection site. Images are from a teratoma containing intestinal epithelium, respiratory epithelium (both endodermal); cartilage, muscle (both mesodermal); neural tube, epidermis (both ectodermal).

To analyze the differentiation potential of these rat iPS cells in vitro, we used floating culture to induce EBs. After suspension culture, ball-shaped EB structures were generated from all rat-iPS clones. We attached these EB-like structures to tissue culture plate and maintained them for 8 to 10 days in ITSFn media [22]. Attached cells showed various types of morphologies, such as those resembling neural cells, muscle cells, and definitive endodermal cells (Fig. 3a). RT-PCR analysis confirmed that these differentiated cells express mRNAs for forkhead box A2 (FOXA2, endoderm marker), Brachyury (mesoderm marker), and bIII-tubulin (Tuj1, ectoderm marker) (Figure S7a). In addition, immunocytochemistry analyses identified cells positive for SRY-box containing gene 17 (SOX17, endoderm marker), α-smooth muscle actin (α-SMA, mesoderm marker), Desmin (mesoderm marker), glial fibrillary alkaline phospatase (GFAP, ectoderm marker) and Tuj1 (ectoderm marker) (Fig. 3a, Figure S7b). Taken together, our gene expression analyses and immunostaining assays of in vitro differentiated cells demonstrate that all rNP-iPS and REF-iPS cell lines tested here are pluripotent and are able to differentiate into all three germ lineage cells in vitro.

To test in vivo pluripotency, we transplanted two rNP-iPS clones (#2 and #4) and two REF-iPS clones (#3 and #4) into the kidney capsule of nude mice. In all four cases, teratoma formation was observed in the kidney capsule at four to six weeks after injection (Fig. 3b, Figure S7c). These rat-iPS-derived teratoma were further analyzed by histological examination and found to contain all three germ layer tissues including pigment retinal epithelium (ectoderm), epidermal tissues (ectoderm), neural tube (ectoderm), cartilage (mesodermal), striated muscle (mesoderm), adipose tissue (mesoderm), intestinal epithelium (endoderm), respiratory epithelium (endoderm) and cornea-like epithelial tissues (endoderm) (Fig. 3b, Figure S7c; data not shown).

### Rat iPS cells can efficiently differentiate into dopaminergic neurons in vitro

**Figure 4:**
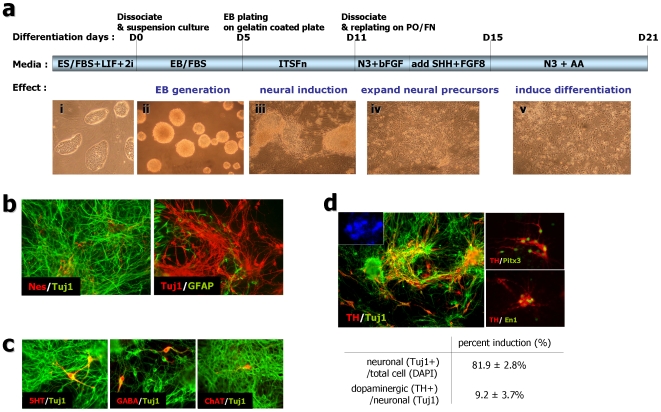
Rat fibroblast derived-iPS cells (REF-iPS#2 cells) progressively induced into dopaminergic neurons. (a) Scheme of the rat iPS differentiation method used to induce dopaminergic neurons from rat iPS cells. Bottom pictures (i∼v) show representative image of each stage. (b) The five-stage neuronal differentiation method induced diverse neural differentiation in rat iPS cells. Six days after withdrawal of the mitogen bFGF at day 21 of the differentiation procedure, expression of nestin (b, left, red), Tuj1 (b, left, green; b, right, red) and GFAP (b, right, green) confirmed the neural identity of the REF-iPS derived neural differentiation. (c) Diverse subtypic neurons are expressed during rat iPS derived neuronal induction. Serotonergic (5-HT, red, left), GABAnergic (GABA, red, middle) or cholinergic (ChAT, red, right)/Tuj1 (green)-positive neurons were induced. Images were taken from REF-iPS#2 derived neuronal cells at day 21. (d) Dopaminergic neuronal differentiation from rat iPS#2-derived neuronal induction at day 21. Representative images of TH (red)/Tuj1 (green)-positive neurons derived from rat iPS cells (Inset, DAPI nuclear staining of the same field). The bottom table shows dopaminergic neuronal differentiation efficiency of rat fibroblast derived iPS cells (REF-iPS #2; from nine independent experiments, mean ± S.E). Indicated are the percent of neuronal (Tuj1+) cells per total (DAPI) cells or dopaminergic (TH+) cells per neurons (Tuj1+) at neuronal induction day 21.

We examined the differentiation potential of rNP-iPS and REF-iPS cells to specialized neural lineage cell types including dopaminergic neuronal fate, based on the five stage in vitro differentiation method [22] with minor modification (Fig. 4a). Six days following withdrawal of the mitogen bFGF (at day 21 in Fig. 4a), immunocytochemistry analysis demonstrated that these rat iPS cells could differentiate into all neural lineage phenotypes, as examined by specific markers for NPs (nestin), neurons (Tuj1), or astrocytes (GFAP) (Fig. 4b). Various subtype neuronal markers were detected for dopaminergic (tyrosine hydroxylase; TH), serotonergic (5HT), GABAergic (GABA) and cholinergic neurons (choline acetyltransferase; ChAT) (Fig. 4b, c). In particular, we found that in vitro differentiation of REF-iPS#2 cells in the presence of sonic hedgehog (SHH), FGF8, and ascorbic acid resulted in high yields of neurons (81.9±2.8% of total cells) and dopaminergic neurons (9.2±3.7% of neurons) (Fig. 4d). In addition, we confirmed that other rat iPS clones (e.g., rNP-iPS #4 and REF-iPS #2 and 3) also showed efficient in vitro differentiation to dopaminergic neurons (data not shown). Furthermore, these TH-positive neurons appear to have midbrain dopaminergic phenotype because they co-express midbrain-specific transcription factors, Ptx3 and En-1 (Fig. 4c).

The rat animal model is a critical experimental system for disease modeling, physiological, pharmacological, and behavioral studies of human [26]. Availability of germ line competent rat ES and iPS cells will greatly facilitate and expand the usefulness of the rat animal model for biomedical and translational research. Here, we established an efficient procedure to generate rat iPS cells from primary rat tissues. Our results demonstrate that iPS cells can be reliably generated without drug selection from primary rat tissues (both NP and REF) by retroviral transduction of the five (Oct4, Sox2, Klf4, c-Myc, and Nanog: OSKMN), four (OSKM), or three (OSK) reprogramming factor genes.

Salient features of this study are as follows. First, our optimized procedure generated multiple rat iPS clones from both NP and embryonic fibroblasts with similar efficiencies without genetic selection suggesting that it may be generally applicable to diverse rat primary tissues. Second, as shown in recent studies to derive rat ESCs [14], [15], inhibition of differentiation-inducing signals, MEK and GSK3β, with chemical inhibitors PD0325901 and CHIR99021, was critical for stable maintenance of rat iPS clones. In addition, LIF was also necessary for rat iPS cell maintenance; rat iPS cells spontaneously differentiated without it even in the presence of PD0325901 and CHIR99021. Third, we found that REF cells were significantly superior to MEF for stable maintenance of rat iPS clones. When maintained on MEF, rat iPS cells significantly lost their ES-like morphology, even in the presence of differentiation signal inhibitors, PD0325901 and CHIR99021, and LIF. In contrast, rat iPS cells could be stably maintained on REF feeders in the presence of two inhibitors and LIF. Li et al. (2008) found that rat ESCs could be stably maintained as adherent cells, without differentiation, when grown on L cells derived from adult C3H/An mouse connective tissues. It will be of great interest to know if rat ESCs can be stably maintained on REFs. Given that both mouse and rat iPS cells are shown to be stably maintained on feeder cells originated from the same species, as shown in mouse [2] and rat iPS cells (this study), it will be interesting to learn whether human iPS cells can also be maintained on appropriate human feeder cells. Indeed, a recent study by Yamanaka and his colleagues demonstrated that human iPS cells can be established and maintained on isogenic parental feeder layers [27]. Forth, our results demonstrate that multiple rat iPS cell lines can be generated from both NP and embryonic fibroblasts by three (OSK) genes without c-Myc although the efficiency was approximately half of that by the four genes. Previous work showed that four factor-derived mouse iPS cells can cause tumor formation in chimeras and progeny mice upon reactivation of c-Myc [28]. Furthermore, iPS cells derived without c-Myc did not develop tumors, strongly suggesting that the presence of c-Myc gene is a serious problem for their biomedical and clinical application. Thus, our successful generation and characterization of cMyc-free rat iPS cells may be important for the future application of rat iPS cells. Fifth, we optimized and established an in vitro differentiation procedure allowing efficient in vitro neurogenesis such that the majority of the differentiated cells become neurons (>80% of total cells) and midbrain-like dopamine neurons can be produced from these rat iPS cells with high efficiency (approximately 10% of total neurons).

Based on previous studies showing that somatic cell fusion with ESCs induced epigenetic reprogramming of somatic chromosomes [23], [25], [29], we also tested if it is possible to generate rat iPS cells by introducing ESC-extracts after streptolysin O-mediated permeabilization. We observed partial reprogramming of rat NP cells as indicated by colony formation, Nanog expression in some colonies, and more potent neural differentiation, compared to non-treated cells (Figure S2). However, we failed to generate iPS colonies by this method. Compared to the in vivo cell fusion approach, our in vitro procedure may allow very limited amount of proteins to be transported inside cells, resulting in only partial reprogramming. Interestingly, the combination of ESC-extract treatment and retroviral transduction of reprogramming factors significantly increased the efficiency of generation of iPS-like colonies, indicating that at least some reprogramming factors may be transported into cells in their active forms.

Taken together, our results demonstrate that AP-positive colonies can be efficiently generated from NP and REF cells (approximately 0.2%) and approximately 20% of them became iPS-like colonies with the ESC morphology. Using our optimized procedure, we established 20 rat iPS clones (11 from NP and 9 from REF) and extensively characterized 4 NP-derived and 4 REF-derived clones in this study. Among them, three iPS clones (rNP-iPS#4, REF-iPS#3 and #4) were generated without c-Myc. All eight clones exhibited the molecular and cellular properties of fully reprogrammed iPS cells such as ESC-like morphology, cell proliferation, endogenous gene expression patterns, epigenetic status, in vitro and in vivo pluripotency. These clones were stably maintained for at least 25 passages with normal karyotype. Thus, our results strongly suggest that ESC-like iPS cells can be derived from genetically unmodified rat tissues including NPcells and fibroblasts. We previously presented some results of this study [30], [31]. While this work was in progress, two groups reported successful generation of rat iPS clones using significantly different procedures. Liao et al. (2009) reported derivation of rat iPS cells from primary ear fibroblasts and bone marrow cells by introduction of the four factors (OSMK). Notably, these iPS cells could be generated and maintained without differentiation signal inhibitors and LIF, which is quite different from our results. One possible explanation is that these rat iPS cells may have different cellular properties because they are generated by lentiviral transduction. Consistent with this possibility, this study reported that iPS cells could not be generated by retroviral transduction. In addition, Li et al. (2009) established rat iPS cell lines from a liver progenitor cell line, WB-F344, by retroviral transduction of the same four factors. Similar to our results, this study showed that differentiation signal inhibitors, PD0325901 and CHIR99021, and LIF are essential for the maintenance of rat iPS cells. Taken together with these rat iPS cell derivation reports, our results demonstrate that iPS cells can be efficiently generated and maintained from genetically unmodified various rat tissues with different sets of factors. These rat iPS clones will provide useful research tools to study rat developmental biology and rat-based models of various human diseases such as 6OHDA-lesioned rat model of PD.

## Supporting Information

Figure S1 Neural precursor (NP) cells isolated from rat E14 cortices are homologous cell populations and are actively mitotic. NP cells were plated at 10,000 cells/cm^2^ and expanded 3 days with bFGF (20 ng/ml) prior to immunocytochemical analyses. Left, a representative image of cells that are mostly positive for nestin (NP marker; red). Only a minor fraction of cells (typically <2%) are positive for TuJ1 (neuronal marker; green). Right, nestin-positive NP cells (green) were double labeled with the proliferation marker, Ki67 (red).
(0.22 MB TIF)

Figure S2 Analysis of clones obtained from cortical NP cells by treatment with ESC-extracts. (a) Representative morphology of clones that are obtained at 2 weeks after treatment with ESC-extracts (right) and untreated NP cells (left). (b) RT-PCR analysis of mRNAs isolated from clones that are generated by treatment with ESC extracts and untreated controls. Some of these clones (approximately 20%) expressed Nanog, but none of them expressed Oct4. (c,d) Differentiation potential of these partially reprogrammed clones. NP cells without ESC treatment became almost completely astrogenic and only differentiated to astrocytes (c, left). In contrast, most clones obtained from ESC-extracts treated NPs, differentiated to astrocytes and neurons with comparable efficiencies (c, right). Differentiated cells were analyzed by immunostaining with antibodies against the neuronal marker, Tuj1 (green) and astrocytic marker, GFAP (red). Quantitative analyses of the differentiation potential of clones obtained from treatment with ESC-extracts (d). Results are presented as the mean ± SEM of % GFAP+ and TuJ1+ cells in the total cell population. (n = 15 from three independent experiments, *P<0.001).
(0.38 MB TIF)

Figure S3 Efficiencies of colony formation by feeder variants. Rat NP-derived ESC-like colony formation is influenced by feeder variants, i.e., mouse embryonic fibroblast (MEF) vs. rat embryonic fibroblast (REF) feeders after treatment with the five, four or three factors. Twenty-days post retroviral transduction analysis showing alkaline phosphatase (AP)-positive clones, on MEF (white) and REF (black) feeder (*P<0.001).
(0.05 MB TIF)

Figure S4 (a) Efficiencies of colony formation after treatment with the five, four or three factors from fibroblasts (REF) and neural precursor cells (NP). Twenty-days post retroviral transduction analysis showing alkaline phosphatase (AP)-positive clones, in black and the total numbers of colonies in white. (Each column from n = 12 (FC) or 14 (NSC) of 6 independent experiments, error bars indicate S.E.) (b) Efficiencies of AP-positive clones (black bar) and SSEA1 and AP double positive clones (patterned bar). These clones are derived from 50,000 NPs by treatment with 4 factors (O,S,K,M) or 3 factors (O,S,K) at 20 days post transduction.
(0.06 MB TIF)

Figure S5 Karyotypic analysis of rat neural precursor cells derived iPS clone #4. No karyotypic abnormalities were observed.
(0.09 MB TIF)

Figure S6 (a) Representative image of REF-iPS cells exhibited strong Rex1 (red; middle) activity. (b) Semi-quantitive RT-PCR analysis of endogenous (endo-) and transgenic (trans-) retroviral Oct4, Klf4 and Sox2 expressions in rat-iPS clones derived from rat neural precursor (rNP-iPS #1, 2 and 4) and fibroblast (REF-iPS #1, 3 and 4). All lines were at passage 10∼14. Expression of endogenous ES marker gene, Rex1, was used as control.
(0.17 MB TIF)

Figure S7 In-vitro and in-vivo differentiation of rNP-iPS clones (#1∼#4). (a) RT-PCR analysis of embryoid bodies (EBs) for three germ layer differentiation markers, endoderm (Foxa2), mesoderm (Brachyury) and ectoderm (βIII-tubulin, Tuj1). (b) Immunocytochemical analysis for differentiation to the three germ layer was performed 10 days after EB attachment. Sox17 (green, endodemal; left), desmine (green, mesodermal; middle), and GFAP (green, ectodermal; right). Nuclei were stained with DAPI (blue). (c) Teratoma derived from rNP-iPS cells. Hematoxylin and eosin staining of teratoma derived from rNP-iPS cells (#2 and #5). Cells were transplanted into kidney capsule of three SCID mice. A tumor developed from one injection site. Each image shows formed teratoma (up/left), cornea-like epithelium (endodermal; down/left), adipose tissue (mesodermal; up/middle), muscle tissue (mesodermal; down/middle), epidermis (ectodermal; up/right) and pigmented retinal epithelium (ectodermal; down/right).
(0.64 MB TIF)
