# Supplementary figures and images for: Direct Reprogramming of Rat Neural Precursor Cells and Fibroblasts into Pluripotent Stem Cells
Source: PLoS One. 2010 Mar 24;5(3):e9838. doi: 10.1371/journal.pone.0009838 (PMC2844422; doi:10.1371/journal.pone.0009838)

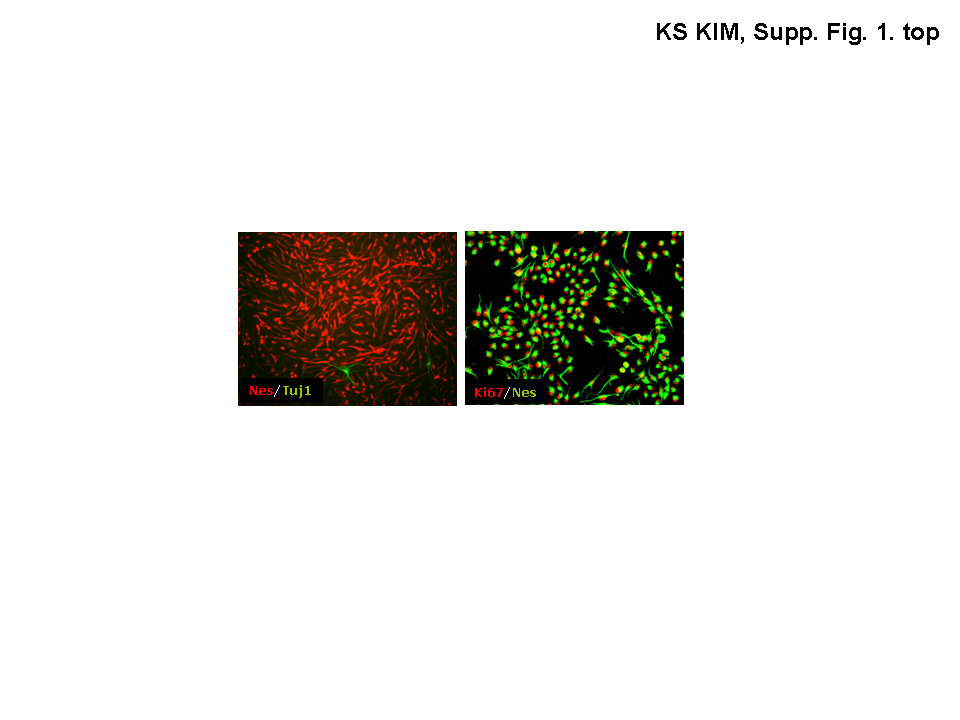

Supplement: Figure S1 — Neural precursor (NP) cells isolated from rat E14 cortices are homologous cell populations and are actively mitotic. NP cells were plated at 10,000 cells/cm2 and expanded 3 days with bFGF (20 ng/ml) prior to immunocytochemical analyses. Left, a representative image of cells that are mostly positive for nestin (NP marker; red). Only a minor fraction of cells (typically <2%) are positive for TuJ1 (neuronal marker; green). Right, nestin-positive NP cells (green) were double labeled with the proliferation marker, Ki67 (red). (0.22 MB TIF) [file pone.0009838.s001.tif]

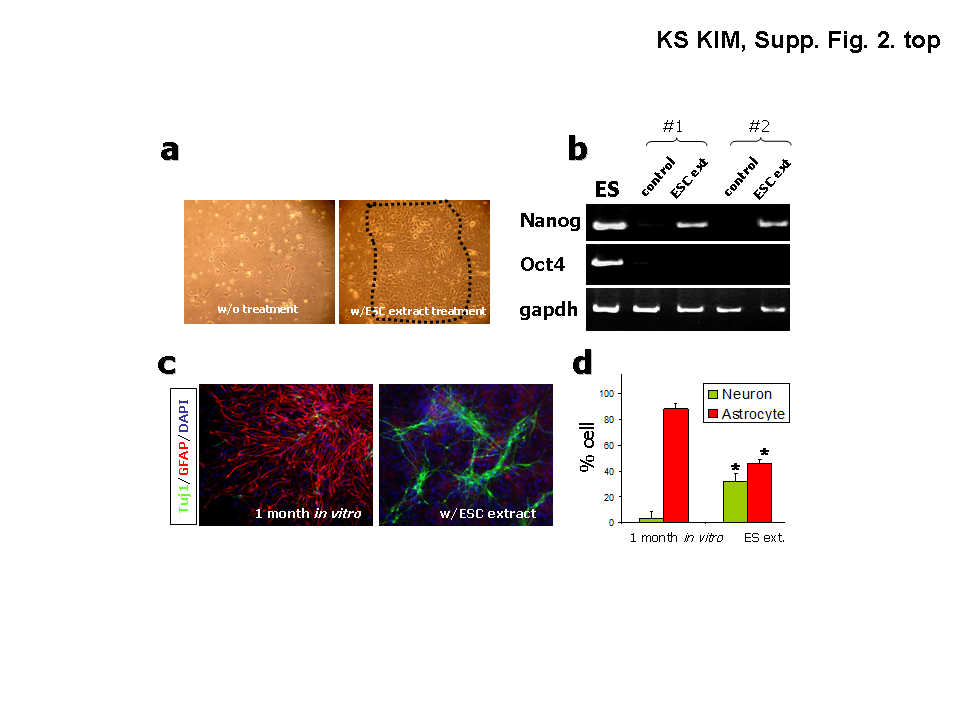

Supplement: Figure S2 — Analysis of clones obtained from cortical NP cells by treatment with ESC-extracts. (a) Representative morphology of clones that are obtained at 2 weeks after treatment with ESC-extracts (right) and untreated NP cells (left). (b) RT-PCR analysis of mRNAs isolated from clones that are generated by treatment with ESC extracts and untreated controls. Some of these clones (approximately 20%) expressed Nanog, but none of them expressed Oct4. (c,d) Differentiation potential of these partially reprogrammed clones. NP cells without ESC treatment became almost completely astrogenic and only differentiated to astrocytes (c, left). In contrast, most clones obtained from ESC-extracts treated NPs, differentiated to astrocytes and neurons with comparable efficiencies (c, right). Differentiated cells were analyzed by immunostaining with antibodies against the neuronal marker, Tuj1 (green) and astrocytic marker, GFAP (red). Quantitative analyses of the differentiation potential of clones obtained from treatment with ESC-extracts (d). Results are presented as the mean ± SEM of % GFAP+ and TuJ1+ cells in the total cell population. (n = 15 from three independent experiments, *P<0.001). (0.38 MB TIF) [file pone.0009838.s002.tif]

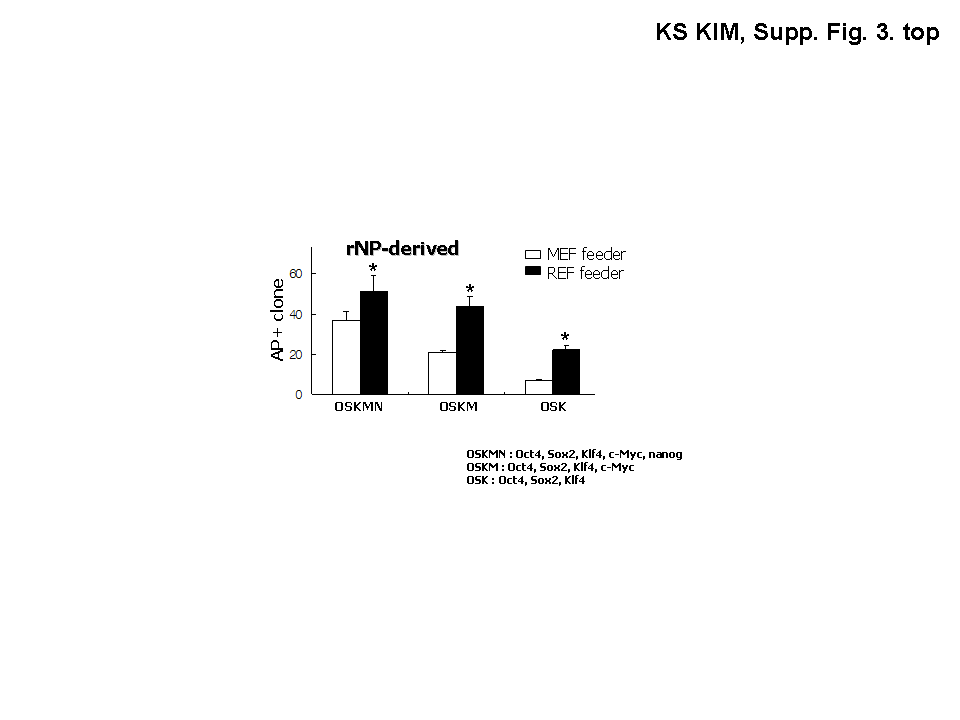

Supplement: Figure S3 — Efficiencies of colony formation by feeder variants. Rat NP-derived ESC-like colony formation is influenced by feeder variants, i.e., mouse embryonic fibroblast (MEF) vs. rat embryonic fibroblast (REF) feeders after treatment with the five, four or three factors. Twenty-days post retroviral transduction analysis showing alkaline phosphatase (AP)-positive clones, on MEF (white) and REF (black) feeder (*P<0.001). (0.05 MB TIF) [file pone.0009838.s003.tif]

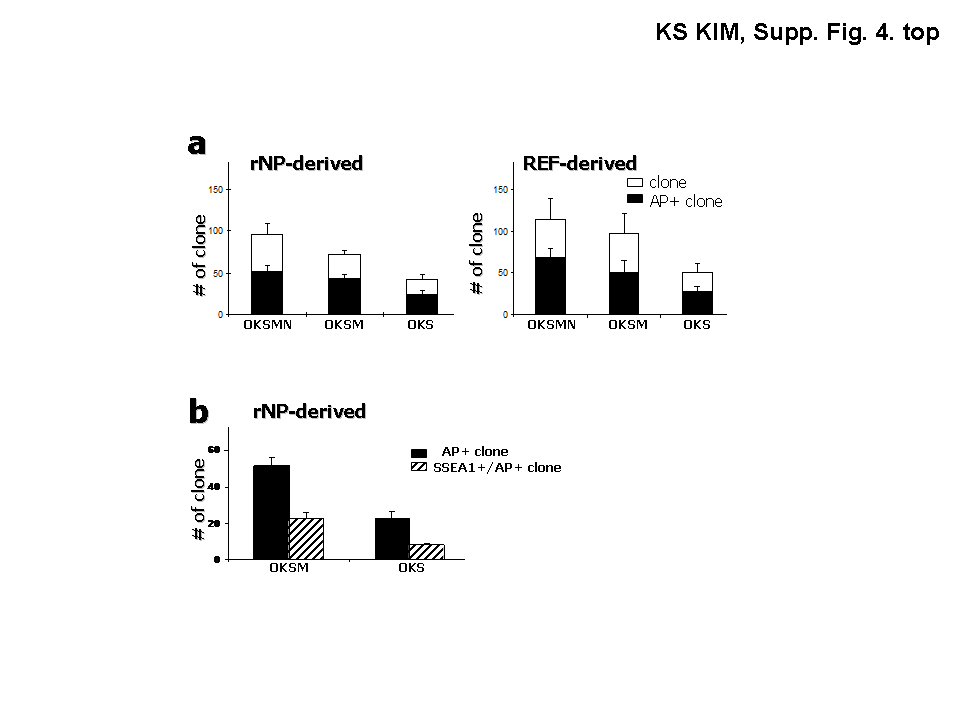

Supplement: Figure S4 — (a) Efficiencies of colony formation after treatment with the five, four or three factors from fibroblasts (REF) and neural precursor cells (NP). Twenty-days post retroviral transduction analysis showing alkaline phosphatase (AP)-positive clones, in black and the total numbers of colonies in white. (Each column from n = 12 (FC) or 14 (NSC) of 6 independent experiments, error bars indicate S.E.) (b) Efficiencies of AP-positive clones (black bar) and SSEA1 and AP double positive clones (patterned bar). These clones are derived from 50,000 NPs by treatment with 4 factors (O,S,K,M) or 3 factors (O,S,K) at 20 days post transduction. (0.06 MB TIF) [file pone.0009838.s004.tif]

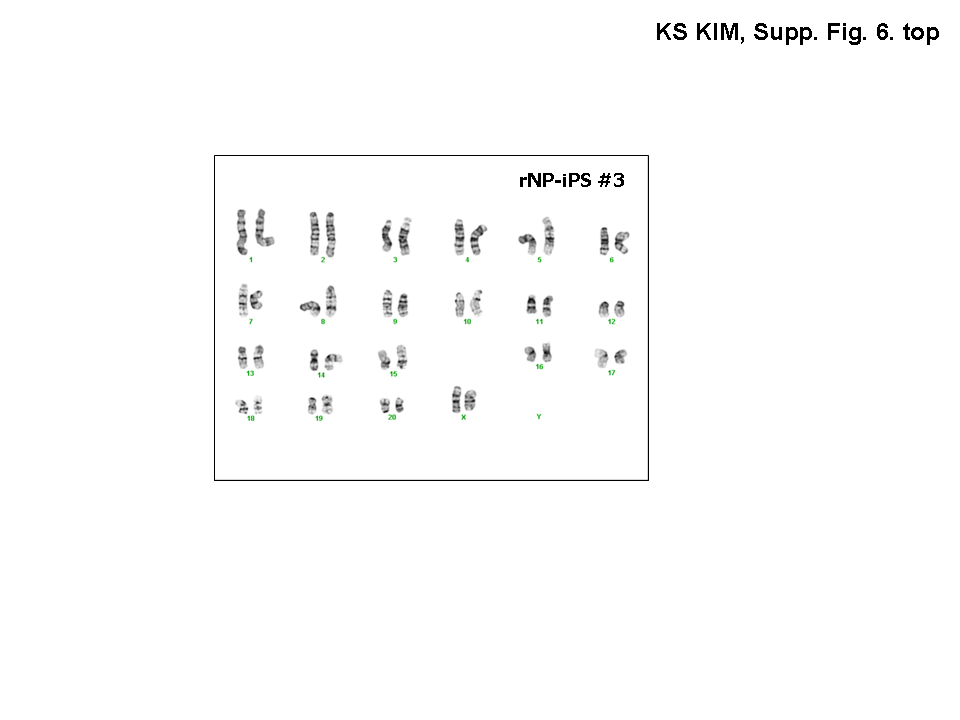

Supplement: Figure S5 — Karyotypic analysis of rat neural precursor cells derived iPS clone #4. No karyotypic abnormalities were observed. (0.09 MB TIF) [file pone.0009838.s005.tif]

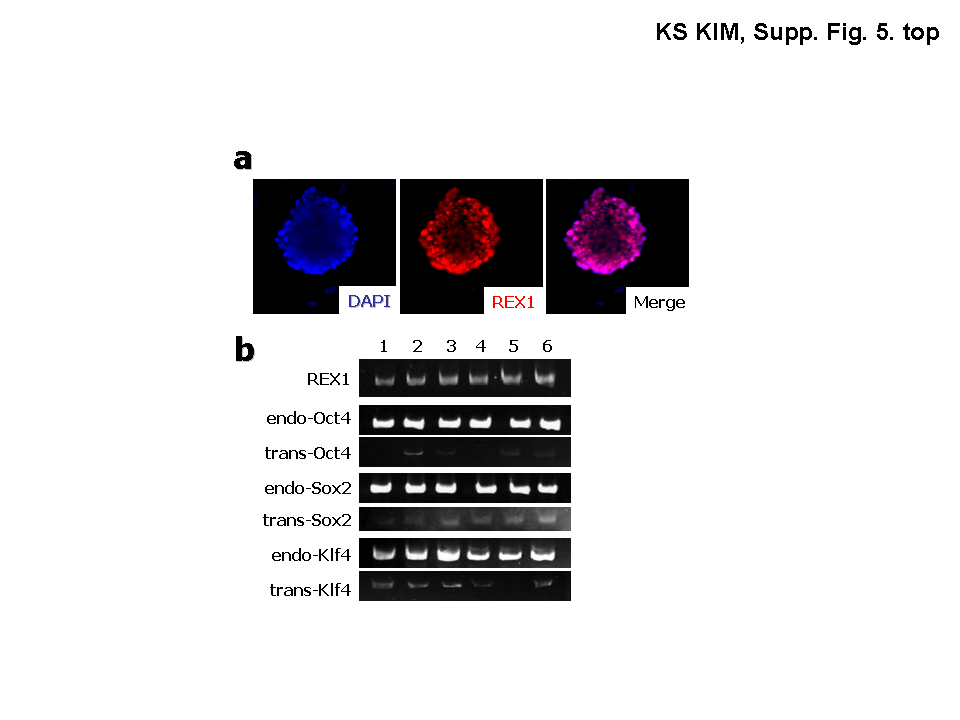

Supplement: Figure S6 — (a) Representative image of REF-iPS cells exhibited strong Rex1 (red; middle) activity. (b) Semi-quantitive RT-PCR analysis of endogenous (endo-) and transgenic (trans-) retroviral Oct4, Klf4 and Sox2 expressions in rat-iPS clones derived from rat neural precursor (rNP-iPS #1, 2 and 4) and fibroblast (REF-iPS #1, 3 and 4). All lines were at passage 10∼14. Expression of endogenous ES marker gene, Rex1, was used as control. (0.17 MB TIF) [file pone.0009838.s006.tif]

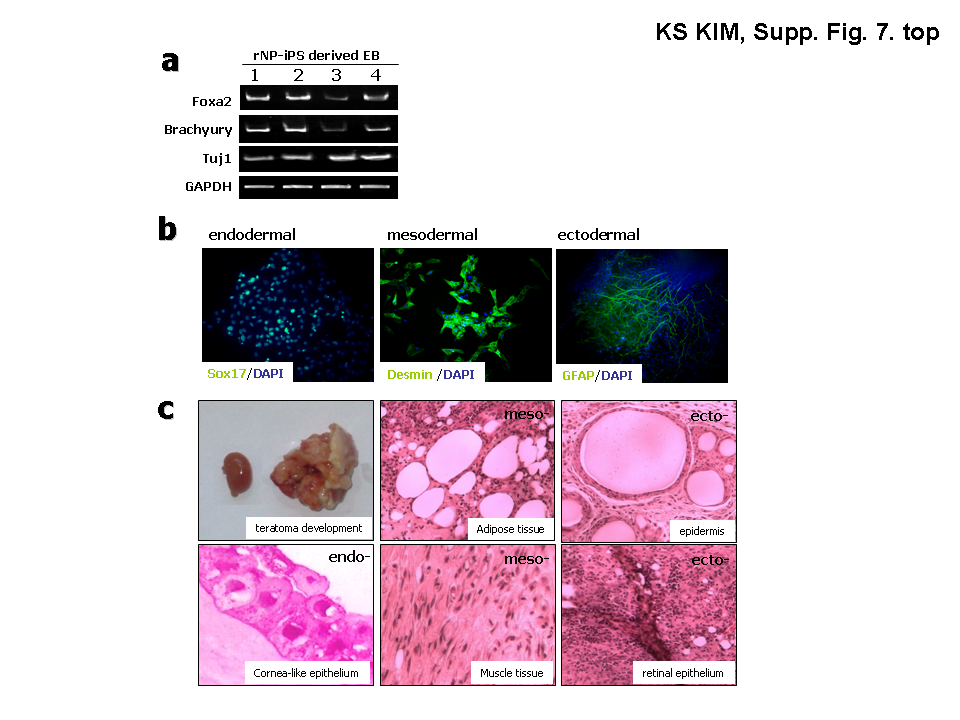

Supplement: Figure S7 — In-vitro and in-vivo differentiation of rNP-iPS clones (#1∼#4). (a) RT-PCR analysis of embryoid bodies (EBs) for three germ layer differentiation markers, endoderm (Foxa2), mesoderm (Brachyury) and ectoderm (βIII-tubulin, Tuj1). (b) Immunocytochemical analysis for differentiation to the three germ layer was performed 10 days after EB attachment. Sox17 (green, endodemal; left), desmine (green, mesodermal; middle), and GFAP (green, ectodermal; right). Nuclei were stained with DAPI (blue). (c) Teratoma derived from rNP-iPS cells. Hematoxylin and eosin staining of teratoma derived from rNP-iPS cells (#2 and #5). Cells were transplanted into kidney capsule of three SCID mice. A tumor developed from one injection site. Each image shows formed teratoma (up/left), cornea-like epithelium (endodermal; down/left), adipose tissue (mesodermal; up/middle), muscle tissue (mesodermal; down/middle), epidermis (ectodermal; up/right) and pigmented retinal epithelium (ectodermal; down/right). (0.64 MB TIF) [file pone.0009838.s007.tif]
